# A novel surgical technique for punctal stenosis: placement of three interrupted sutures after rectangular three-snip punctoplasty

**DOI:** 10.1186/s12886-018-0733-2

**Published:** 2018-03-05

**Authors:** Seong Jun Park, Ju Hee Noh, Ki Bum Park, Sun Young Jang, Jong Won Lee

**Affiliations:** 1 College of medicine, Soonchunhyang University, 204-ho, 31 Soonchunhyang-6-gil, Dongnam-gu, Cheonan, 31151 Choongcheongnam-do South Korea; 2 Soo Eye Clinics, 202-13, Miadong, Kangbook-gu, Seoul, 01118 South Korea; 3 Department of Ophthalmology, Soonchunhyang University Bucheon Hospital, Soonchunhyang University College of Medicine, 170 Jomaru-ro, Wonmi-gu, Bucheon, 14584 Gyeonggi-do South Korea

**Keywords:** Punctal stenosis, Rectangular three-snip punctoplasty, Three interrupted sutures

## Abstract

**Background:**

We developed a novel surgical technique to treat punctal stenosis involving the placement of three interrupted sutures after rectangular three-snip punctoplasty (TSP).

**Methods:**

Retrospective chart review of forty-eight eyes of 44 patients who underwent rectangular TSP with three interrupted sutures was performed. We investigated whether anatomical recurrences (re-stenosis) occurred during the follow-up period. The subjective symptoms of patients were surveyed.

**Results:**

The mean patient age was 64.1 years, and the mean follow-up time was 17.4 months. The placement of three interrupted sutures after rectangular TSP afforded satisfactory outcomes. Regarding subjective symptoms, 91.7% of the eyes (44/48) were reported as improved. Among 4 eyes determined as symptomatic failure, anatomical recurrence (re-stenosis of the punctum) was observed in only one eye. The other three (6.25%, 3/48 eyes) showed functional nasolacrimal obstruction, namely epiphora with patent tear duct.

**Conclusions:**

Placement of three interrupted sutures after rectangular TSP to treat punctal stenosis showed promising results. Notably anatomical success rate was about 98%. Further comparisons between the novel surgical technique and conventional techniques are required.

## Background

Punctal stenosis triggers epiphora in up to 94% of patients [1]. One-snip punctoplasty was first introduced by Bowman in 1853; 164 years later, the search for an optimal surgical procedure resolving punctal stenosis continues [2].

Of the various surgical techniques, three-snip punctoplasty (TSP) has been the most successful method used to enlarge a narrowed punctum [3]. There are two types of TSP, triangular TSP and rectangular TSP, reflecting the shapes of the flaps fashioned during the procedures. Conventional triangular TSP features removal of a triangular flap created by first cutting the vertical canaliculus then the horizontal canaliculus and finally, the base of the canaliculus. Rectangular TSP (also termed posterior ampullectomy) features two snips in the vertical canaliculus and a final snip at the base, with removal of the posterior wall of the ampulla [3]. It has been suggested that sparing of the horizontal canaliculus may ensure preservation of lacrimal physiology [4]. However, this hypothesis has not been confirmed. Although rectangular TSP is associated with a slightly higher resolution rate than triangular TSP (90% vs. 83%), symptom resolution after triangular and rectangular TSP did not differ significantly [3]. Moreover, in cases of severe punctal stenosis, the punctum could not be dilated to make the two initial vertical cuts. For this reason, Kim et al. [4] introduced a new modification of rectangular TSP; rectangular four-snip punctoplasty. Four-snip punctoplasty features one snip in the vertical canaliculus and a second horizontal cut, followed by a third vertical or horizontal cut, and finally removal of the base of the flap. In Kim et al.’s report, the anatomical success rate was 88.9% at 6 months after surgery.

In addition to advances in the surgical procedure, adjunct therapies have also been developed. These include the use of punctal plugs [5], stenting [6], prescription of mitomycin C [7], and punch punctoplasty with a Kelly punch [8]. It remains unclear whether combinations of stenting or mitomycin C with punctoplasty improve outcomes compared to those afforded by punctoplasty alone; the data are conflicting [7, 9, 10].

Here, we describe a novel surgical technique. After rectangular TSP, we placed three interrupted sutures in the posterior wall of the ampulla to maintain punctal enlargement and prevent re-approximation of the cut ends.

## Methods

The study was approved by the Institutional Review Board of Soonchunhyang Bucheon Hospital, Soonchunhyang University College of Medicine, and the study protocol adhered to the tenets of the Declaration of Helsinki.

We placed three ties after rectangular TSP in 48 eyes of 44 patients with punctal stenosis treated at our eye clinics by the same experienced eye surgeon (J.W.L.) from January 2014 to December 2016. This was a non-comparative case series. Patients who had epiphora and severe lower punctal stenosis without nasolacrimal duct obstruction were included. Patients with other causes of epiphora, such as dry eye syndrome, lid laxity, entropion, and ectropion were excluded.

Surgical outcomes were evaluated both subjectively and objectively. Tear meniscus height (TMH) was measured using a slit lamp and changes in tear film volume after surgery were noted [11, 12] . Subjective symptoms of patients were surveyed as improved or not improved. We also noted all recurrences. Anatomical recurrence was assessed based on anatomic re-stenosis.

### Surgical technique

**Fig. 1:**
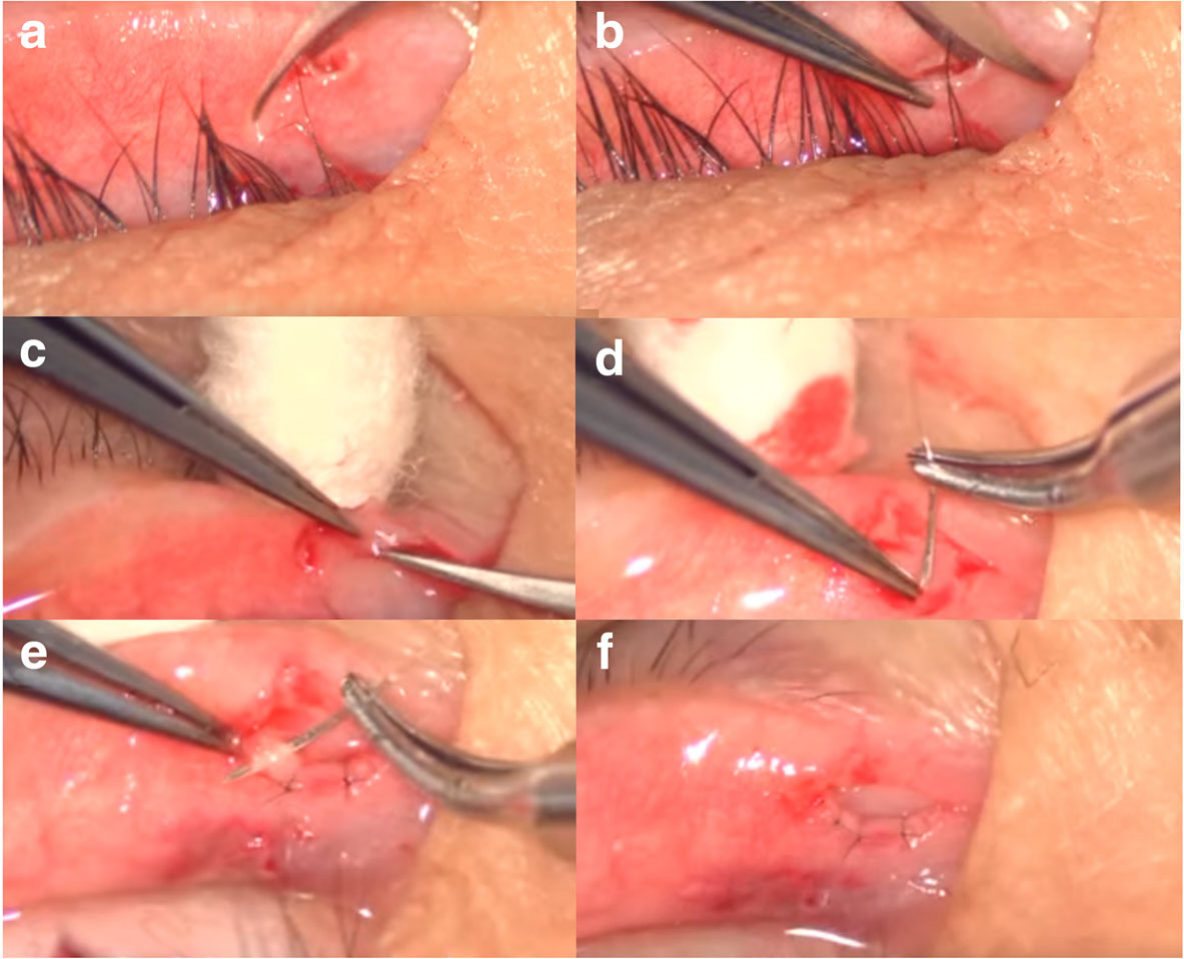
Surgical procedure for the placement of three ties after rectangular three-snip punctoplasty. **a** The first vertical snip at the vertical canaliculus, (**b**) the second vertical snip made from the edge of the first snip to create a flap, (**c**) the final horizontal snip at the base, and (**d**) the three interrupted sutures being placed at the posterior wall of the ampulla after the rectangular three-snip punctoplasty. **e** For the three sutures, the inner surface of the canaliculus was slightly everted to allow sewing to the edge of the tarsal conjunctiva. **f** Immediate postoperative photograph. Note the three interrupted sutures, which are placed at the posterior wall of the ampulla

Surgery was performed using an operating microscope under local anaesthesia (Fig. 1). We transconjunctivally infiltrated 2% (*w*/*v*) lidocaine (with epinephrine in a 1:100,000 weight ratio) from the posterior aspect of the eyelid into the region of the lacrimal canaliculus and punctum. A dilator or small Westcott spring scissors was used to enlarge the stenotic lacrimal punctum. A single blade of a small Westcott spring scissors was placed within the ampulla of the lacrimal canaliculus, with the remaining blade placed on the conjunctival surface of the posterior aspect of the eyelid. The first vertical snip was made at the vertical canaliculus (Fig. 1a). The second vertical snip was made from the edge of the first snip to create a flap (Fig. 1b). The final horizontal snip was made at the base (Fig. 1c). The rectangular flap was removed and three sutures were placed, in an interrupted manner, at the posterior wall of the ampulla using 10–0 nylon (Fig. 1d-f). After the suture was completed, the inner surface of the canaliculus was slightly everted to allow sewing to the edge of the tarsal conjunctiva. The sutures were removed 1 week after the surgery. Levofloxacin and fluorometholone eye drops were used q.i.d. for 1 week.

### Statistical analysis

Data were analysed with the aid of the Statistical Package for the Social Sciences (SPSS) version 20.0 (SPSS INC., Chicago, IL, USA). The paired t-test was used to compare mean TMH values before and after the procedure. A *p*-value < 0.05 was considered to reflect statistical significance.

## Results

Forty-eight eyes of 44 patients were enrolled. The mean patient age was 64.1 years. Ten patients were male and 34 were female. The average follow-up time was 17.4 months. The causes of punctal stenosis were idiopathic (35 patients), severe viral keratoconjunctivitis (six patients), ocular pemphigoid (two patients), and systematic chemotherapy (one patient).

**Fig. 2:**
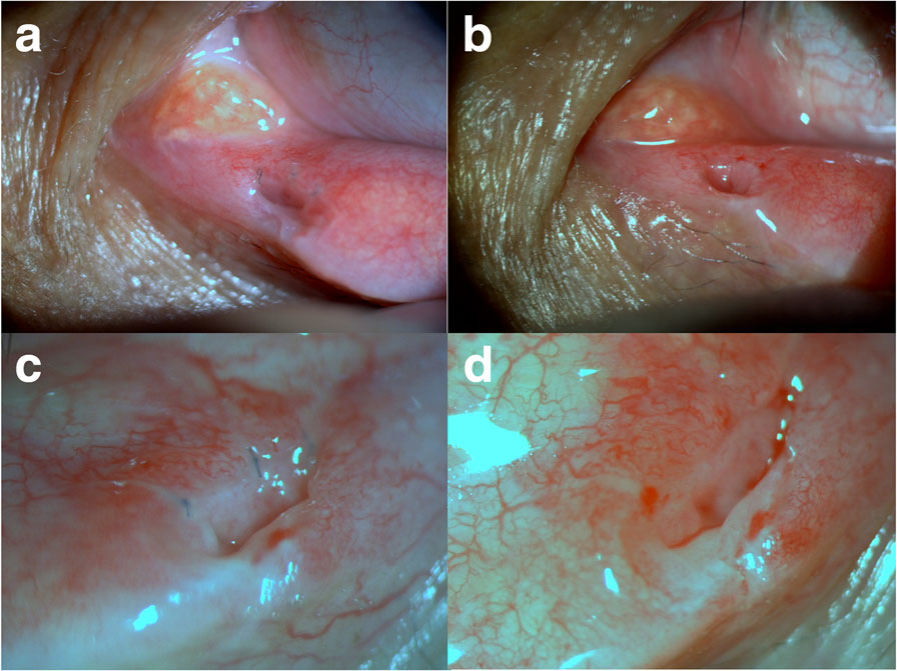
Pre- and post-operative representative photographs of punctoplasty. Postoperative photographs of a 65-year-old female at (**a**) 1 week and (**b**) 1 month. Note the enlarged punctal opening. Postoperative photographs at (**c**) 1 week and (**d**) 1 month of the right lower eyelid punctum of a 63-year-old male

The placement of three ties after rectangular TSP afforded both subjective and objective improvements. The surgery required 5–10 min. for one case. The patient subjective symptom survey showed that 44 eyes (91.7%) were improved, and four eyes (8.3%) remained unchanged. The mean TMH decreased from 1.4 mm (0.5–3 mm) to 0.8 mm (0.5–2 mm). The paired *t*-test revealed a significant difference between the pre- and postoperative TMH (*p* < 0.001). Representative photographs of pre- and post-operative punctoplasty are shown in Fig. 2.

Among four eyes determined as symptomatic failure, anatomical recurrence (restenosis of the punctum) was observed in only one eye (a patient with idiopathic punctal stenosis). Thus, the anatomical recurrence rate was about 2.1% (1/48 eyes) with an average recurrence time of 8.7 months. The other three (6.25%, 3/48eyes) showed functional nasolacrimal obstruction, namely epiphora with patent tear duct, with an average recurrence time of 5.2 months.

## Discussion

In the current study, we placed three interrupted sutures in the posterior wall of the ampulla after rectangular TSP; both the subjective and objective surgical outcomes were satisfactory; 91.7% of eyes were reported improved over about 17.4 months of follow-up. TMH is significantly correlated with the volume of the tear film, of which 75–90% is contained in the tear meniscus [13–18]. We used TMH to measure changes in tear film volumes after surgery. The mean TMH decreased significantly (*p* < 0.001).

Punctal stenosis is the cause of 8% of all epiphora encountered in tertiary care institutions [19]. Punctoplasty is a simple surgical procedure that resolves punctal stenosis. The history of punctoplasty contains many surgical modifications and developments [2]. One-snip punctoplasty was the first modality to be introduced. Problems included deterioration of the capillarity of the lacrimal canaliculus and difficulties in re-approximation of the raw cut ends. Thus, one-snip punctoplasty was replaced by two-snip punctoplasty and TSP [2]. Currently, TSP is the most successful surgical technique used to enlarge a stenosed punctum [3]. There are two types of TSP: triangular TSP and rectangular TSP, the latter is believed to afford better symptom resolution [2, 3]. However, rectangular TSP is still associated with a high recurrence rate of functional epiphora (10.3%) [1]. Thus, efforts towards an optimal surgical procedure for resolution of punctal stenosis continue.

The anatomical recurrence rate of this study was about 2.1% with an average follow-up time of 17.4 months. Chak and Irvine reported that the anatomical recurrence rate was about 6% (3/49 eyes) after conventional rectangular TSP and about 3% (2/50 eyes) after conventional triangular TSP [3]. Ali et al. [1] recently reported an anatomical recurrence rate of 5.7% after conventional rectangular TSP; this value was similar to that of Chak and Irvine [3]. Our anatomical recurrence rate was lower and our average follow-up time was 17.4 months, which was longer compared with 8.2 months in the study of Chak and Irvine [3] and 4.2 months in the study by Ali et al. [1]. It is promising that our new technique shows a lower recurrence rate than conventional rectangular TSP of the two cited studies, despite having a longer follow-up period. In this study, the functional recurrence rate was about 6.25%, which was higher than the anatomical recurrence rate. Recently, long-term outcomes of punch punctoplasty using a Kelly punch have been reported with promising results [8]. In this report, the anatomical success was 94% and the functional success rate 92%.

Our rationale to explain the promising results of the three sutures after rectangular TSP was that the interrupted sutures helped to decrease the raw surface of the dilated punctum and making restenosis of the dilated punctum less likely.

Although the invasiveness of the new procedure still remains a concern, our new technique involving the placement of three sutures after rectangular TSP has shown promising results. Physiological preservation of the lacrimal system should be further reviewed in comparison with that provided by conventional rectangular or triangular TSP as control groups. A limitation of this study was a lack of preoperative grading of stenosed puncta.

## Conclusions

Punctal stenosis commonly triggers epiphora requiring surgical intervention. Among various punctoplasty procedures, three-snip punctoplasty is the most successful surgical technique when it is necessary to enlarge the punctum in patients with punctal stenosis. In this study, we introduce a novel surgical technique involving placement of three interrupted sutures in the posterior wall of the ampulla after triangular three-snip punctoplasty. The new surgical procedure has shown promising results in punctal stenotic patients and may increase the success rate of punctoplasty.

## Abbreviations

TMH: Tear meniscus height
TSP: Three-snip punctoplasty
